# Some vexations that challenge viral immunology

**DOI:** 10.12688/f1000research.8391.1

**Published:** 2016-05-27

**Authors:** Barry T. Rouse, Scott N. Mueller

**Affiliations:** 1 Department of Pathobiology, College of Veterinary Medicine, University of Tennessee, Knoxville, TN, USA; 2 Department of Microbiology and Immunology, Peter Doherty Institute for Infection and Immunity, The University of Melbourne, Victoria, Australia

**Keywords:** viral immunology, vaccine, immunomodulators, immunology, acquired immunity

## Abstract

The field of viral immunology seeks to understand mechanisms of virus-host interaction with a view of applying this knowledge to the design of effective vaccines and immunomodulators that control viral infections. This brief review discusses several areas of the field that hold substantial promise for translation, but where further work is critically required to find solutions. We emphasize that our fundamental understanding of virus-host relationships is moving in leaps and bounds, but we lag behind in applying this knowledge to the successful control of many viral infections.

## Introduction

The science of immunology has its roots in infectious disease, with its pioneers striving to explain how a body defends itself against infections and why vaccines protect in many, although not in all, instances. Few immunologists can claim to have applied the fundamental understanding of their science to develop effective new vaccines, but this has always been the expectation. In fact, the victory of vaccines (almost all developed empirically) is complete for some agents but is notably absent for many others. Immunologists might believe that once all the mechanistic facts are in and digested, it is just a matter of time before strategies will be designed to deal with current vexations in infectious disease, autoimmunity, allergy and asthma, transplantation, cancer, and even subtle problems that affect the nervous system. This optimism is based on the enormous accumulation of data, the availability of many powerful *in vivo* models and *in vitro* test systems, the enthusiasm, confidence, and energy of researchers, and, yes, the abundance of funding from both government sources and private philanthropists. However, translation of such abundant and complex experimental data into a sufficiently deep understanding of the biology of the immune system that will permit the design of effective vaccines against what are currently elusive targets (i.e. HIV) is a goal we are yet to reach. In this brief review, we discuss some topics in the field of viral immunology that remain problematic but could be resolved as a consequence of accumulating new data and ideas. We expect to encourage argument and hope to inspire solutions.

## How should we deal with viruses that lack effective vaccines?

This question becomes more pressing with agents that persist and cause chronic lesions, but new pathogens are always emerging (for example Ebola, or in recent years the Zika virus). Moreover, several commonly occurring acute viral infections also lack effective vaccines. A significant example is respiratory syncytial virus (RSV), a frequent cause of respiratory disease in infants, especially those born prematurely. Vaccination in early childhood is often not successful due to poor adaptive immunity and Th2-skewed responses. Progress in vaccine development for RSV has been slow for several reasons. A vaccine produced several years ago proved highly unsatisfactory, since this inactivated vaccine caused some recipients to express enhanced disease when exposed to natural infection, likely because the vaccinees developed immunopathological reactions ^1^. Other reasons for slow vaccine development include the lack of ideal animal models to study RSV pathogenesis and the fact that natural infection of infants does not make them immune to reinfection because of their immature immune systems. However, hope for success recently came from structural studies on the F protein, a target for neutralizing antibodies needed to protect against RSV ^1^. It was shown that neutralizing antibody-inducing immunogenic epitopes could be expressed on the RSV F protein if it was reengineered to prevent the loss of neutralizing antibody stimulating epitope expression, as happens normally when the native F protein fuses with the cell the virus infects ^1^. We anticipate that structural biologists might provide insights about ligand-antibody complexes for other viral proteins, such as with influenza and HIV, and this will result in the design of immunoprotective vaccine formulations. This topic was recently reviewed ^2^.

With regard to persistent pathogens, two of the most troublesome chronic viral infections that lack vaccines are HIV and hepatitis C virus (HCV). Fortunately, both viruses can be effectively controlled by combination drug therapy, but this is not an ideal solution and is prohibitively expensive in the case of HCV. For these two diseases, prophylactic as well as therapeutic vaccines are needed. Other chronic infections, such as hepatitis B virus (HBV) and human papilloma viruses (HPVs), do have effective prophylactic vaccines, but there is a need for therapeutic vaccines for those already infected ^3^. However, producing effective therapeutic vaccines against any infection provides a major challenge.

The search for a vaccine against HIV has been vigorously pursued since the virus was first identified in the early 1980s. We have experienced periodic occasions of apparent success ^4^, but none have withstood the scrutiny of independent verification. However, some evidence inspires optimism that an effective vaccine could eventually be produced. It is well known that some infected persons who are not receiving therapy successfully control the infection for prolonged periods of time ^5^. Such elite controllers indicate that a protective immune response can occur, although defining the immunological signature of control and, importantly, duplicating it with a vaccine has proven elusive. Moreover, elite controllers do not eliminate the virus from their system and some patients ultimately lose elite status likely because of the eventual emergence of virus variants that manage to escape the determined efforts of the immune system.

Optimism for an eventual effective HIV vaccine comes from animal model studies on lentivirus infections in primates ^6^. Many different approaches have been explored and shown promise, but none more so than that pioneered by Louis Picker and colleagues ^7^. This group showed that engrafting selected simian immunodeficiency virus (SIV) proteins into a rhesus cytomegalovirus gene modified vector (Rh-CMV) induced a high level of protective immunity which in many cases fully controlled virus replication upon challenge with a highly virulent SHIV challenge ^7^. The success of this form of immunization was likely explained by a very broadly reactive and unusual CD8 ^+^ T cell response that was largely composed of CD8 ^+^ T cells that recognize peptides presented by class II major histocompatibility complexes (MHCs) rather than the normal class I MHC restricting elements. These CD8 ^+^ class II restricted responses were unique and did not overlap with the conventional CD8 ^+^ responses. The latter dominate when animals were vaccinated with other types of vaccines. The Picker results are highly encouraging and were duplicated using Rh-CMV vectors containing some other antigens, most notably *Mycobacterium tuberculosis* (not yet published). One hopes the Picker approach is independently confirmed and can be tested to see if a similar protective pattern of responsiveness can be achieved against HIV in humans. Also in need of explanation will be the observation that the Rh-CMV vector vaccination approach was successful in only approximately 50% of animals, yet all developed the predominant non-canonical pattern of CD8 ^+^ T cell responsiveness.

Whereas several viral infections are still in need of successful prophylactic vaccines, in many instances we also require therapeutic vaccines that could boost inadequate immunity. This is a requirement in many chronic infections, which includes almost all herpesvirus infections as well as HIV, HCV, and HBV and those chronically infected with HPV who missed out on the highly effective prophylactic vaccines. There are only a few success stories with therapeutic vaccines, but the one which stands out is the apparent success of varicella zoster vaccine used to diminish the chance of shingles in elderly patients infected decades previously with the chickenpox virus ^8^. However, the need for effective therapeutic vaccines is emphasized in the case of HCV infection. In this instance, 20–30% of persons infected with HCV successfully resolve their infection and usually remain immune to reinfection ^9^. Unfortunately, the majority fail to resolve their infections and these persons undergo gradual deterioration in liver function, which may ultimately fail, or worse still evolve into hepatocellular carcinoma. In the affluent world, chronic HCV can now be controlled using an inexcusably expensive new drug combination therapy ^10^. Nevertheless, once the virus is cleared in those fortunate drug recipients, they remain fully susceptible to reinfection, which in fact often occurs ^10^. Thus, with HCV we need therapeutic vaccines that would protect not only those who are chronically infected but also patients who have undergone a successful form of drug therapy. These issues lead to the general question of whether the balance of immunity can be changed in those situations where it is ineffective or even contributes to tissue damage.

## Manipulating memory responses

A hallmark of acquired immunity is memory, a topic that interests all viral immunologists, especially those striving to improve vaccines or control infections in other ways. Many rules for memory have been established using mouse models, some of which apply and others which may differ somewhat in humans. Memory involves lymphocytes and their derivatives and once a pattern is established, it may be hard to change; certainly in humans, memory can be very long lasting. It is becoming increasingly evident that the components of memory reside in different locations, which in some instances are resistant to relocation ^11^. Moreover, in adult humans, the great majority of memory cells are located at mucosal, skin, or non-lymphoid tissue sites. Indeed, it is now evident that the memory cells most instrumental in providing the first line of protection against pathogens are located at the tissue and mucosal entry sites. We now have a better understanding of how these tissue-resident memory (T _RM_) cells get there and that a genetic program directed in part by the tissues restricts the expression of molecules that would promote their relocation ^11, 12^. However, we need to better understand how T _RM_ cells are regulated in tissues, how they contribute to protective immunity, how long they remain immunoprotective, and if they can be expanded in numbers and function. Clues are emerging on all fronts and the topic has received lucid reviews ^11, 12^. It seems evident that immunity to reinfection, even in those already infected, as occurs with many viruses (particularly RNA viruses but several herpes viruses too), might depend critically on the numbers and function of T _RM_ cells. This was well established with HSV in mice ^13^, but the main practical question is how we can expand and boost local memory in humans. Artificially, this can be achieved in mice by pulling cells into tissue sites by the injection of relevant chemokines ^14^. Another approach might be to cause T _RM_ cells to multiply and become more functional *in situ* perhaps by using appropriate innate immune ligands with or without cocktails of cytokines, such as interleukin (IL)-7 and IL-15. Although the maintenance of T _RM_ cells in mice does not require antigen, it remains possible that the provision of some form of antigen and/or co-stimulators might succeed in expanding T _RM_ populations, presumably via recruitment of more cells into the tissue. However, we need to remain cautious, since recruiting very large numbers of T cells into the tissues could have detrimental effects. These issues are of high importance and are under active investigation by many groups, which includes one of us.

Another important aspect that impacts on immune protection is its potential erosion during persistent infections, commonly referred to as exhaustion ^15^. T cells constantly exposed to antigen show a multi-faceted change to their differentiation state, including a progressive diminishment of effector functions, upregulation of a series of inhibitory receptors (including PD-1, TIM-3, LAG-3, CTLA-4, and others), and altered gene expression and metabolism. This dramatic change in T cell fate negatively impacts memory T cell generation. First characterized during chronic LCMV infection in mice, many persistent human infections and cancers induce this dysfunctional state in responding T cells. Of notable interest for vaccines and therapies, impeding ligand binding to these receptors causes a rebound of T cell activity and better control of infection ^15^. This inhibitor blocking approach, along with the manipulation of other checkpoint controls on T cell function, has found exciting application to the control of some cancers ^16^. Yet it remains to be seen whether such checkpoint blockade could improve outcomes in patients with established chronic infections such as HIV and HCV. Encouragingly, combining checkpoint blockade with therapeutic vaccination to enhance T cell responses and improve viral control has shown promise in models of both HPV and SIV infection ^3, 17^. Indeed, improving T cell functions may help improve therapeutic vaccine efficacy against HCV and perhaps even against herpesviruses.

## Blunting immune-mediated tissue damage

In many virus-induced lesions, the tissue damage results from immunopathology and this is the consequence of an imbalanced response to infection ^18^. Accordingly, control could result if the balance of the response was reprogrammed, although this objective is more feasible during the induction compared to the effector phase of immunity. With regard to rebalancing the pattern of adaptive immunity, several approaches show promise, although most are not antigen specific and hence could have unwanted side effects. The non-specific approaches include using modulators of innate immunity that impact on the pattern of adaptive immune responsiveness ^19^, manipulating the expression of host molecules that put brakes on immune protection, such as galectins and lipid-derived mediators ^20^, or exploiting metabolic differences between cell subsets to selectively expand protective T cells ^21^. Our own work has focused on manipulating the levels and functions of regulatory T (T _reg_) cells in virus-induced tissue damaging lesions, as this approach could conceivably be made antigen specific. Manipulating the T-effector/T _reg_ cell balance is most easily achieved during the induction phase, but the usual clinical demand is to reformulate the pattern of events once an unfavorable profile has been established. A clue for the potential success of reformulating profiles may be the discovery that the function of fully differentiated T cell subsets is plastic and can be reprogrammed in response to ongoing events in their environment ^22^. The focus has been on plasticity changes between pro-inflammatory cells such as Th1 and Th17 CD4 ^+^ T cells in relation to numbers of T _reg_ cells. Understanding and harnessing plasticity to manage immune-mediated autoimmune disease has been championed by the Bluestone group ^22^. In viral-induced immunoinflammatory disease, we usually need to expand T _reg_ cells at the expense of pro-inflammatory T cells. Few practical avenues are available as yet to accomplish reprogramming of fully differentiated T cells *in vivo*, ** but those approaches which do seem promising include the targeting of cytokines such as IL-6, IL-1, and IL-12 that are involved in driving T _reg_ plasticity ^22^. Since plasticity involves changes in epigenetic control, this process can be targeted with drugs that block DNA methyltransferases ^23^ or histone deacetylases ^24^. Other potentially practical approaches are to target downstream signaling events which differ between T _reg_ and T-effector cells. This can be achieved with the drug rapamycin, which inhibits mTORC1. In T effectors, mTORC1 is involved in the production of inflammatory cytokines which will be inhibited by rapamycin. In T _reg_ cells, mTORC1 is not active except when the cells are undergoing plasticity. Hence, rapamycin will not inhibit functional T _reg_ cells and as a bonus will stop the cells from becoming proinflammatory. The outcome will be a change in the overall balance that favors T _reg_ cells ^22^. Perhaps of particular value eventually will be to exploit the known differences in glucose and fatty acid metabolism by T _reg_ and effector T cells ^21^. For example, T _reg_ cells can be expanded based upon their known requirements of high fatty acid oxidation and low glucose consumption. This can be achieved with drugs such as soraphen A, which inhibits fatty acid synthesis ^25^, and 2-deoxyglucose, which blocks glycolysis ^26^.

## How can novel technology lead to making better vaccines?

Vaccines are among the greatest success stories in modern medicine, but in most cases we do not fully understand how host defense mechanisms account for their success. Such information is necessary, since it should help guide vaccine improvements and could reveal how to develop vaccines against the many agents that still lack them. The information needed to design novel effective vaccines is expected to come from the tools elaborated by systems biology, popularly referred to as “omics” ^27^. These approaches are all high throughput and assemble an abundance of data, which include DNA microarrays, proteomics, genomics, transcriptomics, metabolomics, epigenomics, and deep sequencing. The expectation is that this combination of high-throughput data, along with measurements of immunological parameters by conventional immunological assays, could be subjected to complex computational analysis to yield a signature indicative of optimal immunogenicity and vaccine efficacy or failure. Immunological signatures have already been defined for successful vaccines, such as the yellow fever vaccine ^28^. The hope is that signatures should be invaluable to guide the design of more effective vaccines and to evaluate or improve those vaccines already available. However, so far, predictive signatures have had limited success in guiding the development of improved or novel vaccines or explaining why existing vaccines fail in some individuals but not in others. This topic of systems vaccinology enjoys great enthusiasm and support, and exciting discoveries emerge almost daily. For example, as this review was being written, a report provided a molecular signature to explain why some influenza vaccine recipients develop clinical adverse responses ^29^. With regard to the value of systems biology, the future is bright, but meaningful success remains a vexation.

## Can the microbiome explain almost everything?

Few topics currently enjoy more journal space in immunology than the realization that microbes and other residents at mucosal and surface sites can influence systemic immune responses to many antigens that include viral infections and vaccines ^30^. With viral infections, a report by Iwasaki showing that the microbiome of the gut impacted on immunity to influenza in the lung was the beginning ^31^, but many reports along similar lines have followed. Of particular interest to viral immunology is that viruses can influence the outcome of infections to other types of agents. For example, herpesvirus latency can impact on the outcome of infection with Listeria bacteria and parasitic worms ^32^, and norovirus infection influences the outcome of enterococcal infection ^33^. An insightful and thought-provoking review on this topic, which is referred to as transkingdom metagenomics, was published recently by Pfeiffer and Virgin ^34^. The translational application of the field is that manipulating the microbiome (and/or other “omes”) might improve the success of vaccines and perhaps other immunomodulators. However, as argued by Hanage ^35^, the field requires a healthy dose of skepticism. This includes the need to decide between cause and mere correlation, the issue of whether experiments detect differences that matter and reflect reality, as well as the question of whether anything else could explain the findings. On balance, however, we remain optimistic that in the long term translational benefits to the viral immunology field will accrue. Indeed, it could be that bugs will be useful as therapies to manage certain viral infections.

## Some conclusions

The field of viral immunology is alive and well, and researchers continue to make significant and exciting contributions to our understanding of the fundamental biology of the immune system. Yet the practical translational application of this fascinating and fast-moving area of science is just a little disappointing. Some novel vaccine formulations have been developed, but far more are needed. We have also been successful in generating useful immunomodulators, but invariably their cost is prohibitive and beyond the reach of most persons. We have tooled up to respond briskly to new viral emergences such as Ebola and Zika, but in reality making and getting permission to test new vaccines and therapies remains a slow process. We also need to fully douse the idea held by some in society that the effective vaccines we use cause more harm than good. The introduction of “improved” vaccine formulations with fewer side effects may have the unwanted consequences of reduced efficacy. One example of this is the shift from whole-cell to acellular pertussis vaccines, which it now appears are not providing optimal control of pertussis. Moreover, loss of herd immunity within communities in developed countries owing to reduced vaccination rates, as parents and even doctors lose sight of the risks of not vaccinating, remains a clear and present danger. Nevertheless, the tools to develop and design new vaccines and predict efficacy continue to improve as our basic understanding of the immune system advances. As the promise and enthusiasm for new vaccines and drugs continue to drive research funding and public health agendas, the outlook suggests that there is much on the horizon.
